# Androgen deprivation therapy sensitizes triple negative breast cancer cells to immune-mediated lysis through androgen receptor independent modulation of osteoprotegerin

**DOI:** 10.18632/oncotarget.8274

**Published:** 2016-03-22

**Authors:** Anna R. Kwilas, Andressa Ardiani, Sofia R. Gameiro, Jacob Richards, Ashley B. Hall, James W. Hodge

**Affiliations:** ^1^ Laboratory of Tumor Immunology and Biology, Center for Cancer Research, National Cancer Institute, National Institutes of Health, Bethesda, MD, USA

**Keywords:** enzalutamide, abiraterone, androgen deprivation therapy, TNBC, immunogenic modulation

## Abstract

Among breast cancer types, triple-negative breast cancer (TNBC) has the fewest treatment options and the lowest 5-year survival rate. Androgen receptor (AR) inhibition has displayed efficacy against breast cancer preclinically and is currently being examined clinically in AR positive TNBC patients. Androgen deprivation has been shown to induce immunogenic modulation; the alteration of tumor cell phenotype resulting in increased sensitivity to immune-mediated killing. We evaluated the ability of AR inhibition to reduce the growth and improve the immune-mediated killing of breast cancer cells with differing expression of the estrogen receptor and AR. While AR expression was required for the growth inhibitory effects of enzalutamide on breast cancer cells, both enzalutamide and abiraterone improved the sensitivity of breast cancer cells to immune-mediated lysis independent of detectable AR expression. This increase in sensitivity was linked to an increase in cell surface tumor necrosis factor-related apoptosis-inducing ligand (TRAIL) receptor expression as well as a significant reduction in the expression of osteoprotegerin (OPG). The reduction in OPG was further examined and found to be critical for the increase in sensitivity of AR- TNBC cells to immune-mediated killing. The data presented herein further support the use of AR inhibition therapy in the AR+ TNBC setting. These data, however, also support the consideration of AR inhibition therapy for the treatment of AR- TNBC, especially in combination with cancer immunotherapy, providing a potential novel therapeutic option for select patients.

## INTRODUCTION

Breast cancer is routinely categorized based on receptor expression, including estrogen receptor (ER), progesterone receptor (PR) and Her2/neu receptor (Her2) expression. One of the major standard-of-care therapeutics for the treatment of breast cancer is estrogen deprivation therapy, most commonly tamoxifen, for pre-menopausal breast cancer patients, or an aromatase inhibitor for post-menopausal patients [1]. However, the utility of estrogen deprivation therapy is limited to patients with breast cancer that is ER/PR positive (ER/PR+). Ten to twenty percent of patients diagnosed with breast cancer are diagnosed with triple-negative breast cancer (ER-, PR-, Her2-; TNBC) for which the 5-year survival rate is only < 30% [2, 3]. This low 5-year survival rate is partially due to the lack of available targeted therapeutics for this patient population. Recently, studies have sought to further refine the classification of breast cancer, particularly TNBC, through more detailed analysis of gene expression profiling and histopathological features as well as additional receptor expression, in an effort to identify potential new therapeutics for this patient population. This has led to the adoption of 7 subtypes of TNBC as well as identification of the androgen receptor (AR) as a potential prognostic indicator and therapeutic target for these patients [4–12].

The AR is expressed by 12-36% of TNBC [13–15]. In addition, elegant studies have identified that AR positive (AR+) TNBC is dependent on androgens for growth and that inhibition of AR signaling results in growth inhibition of these cells both *in vitro* and *in vivo* [16–18]. Androgen deprivation therapy (ADT) is a standard-of-care for prostate cancer [19]. Enzalutamide and abiraterone are two ADT agents currently approved by the U.S. Food and Drug Administration (FDA) for the management of castration-resistant prostate cancer; previous studies have shown that indeed enzalutamide has growth inhibitory effects on AR+ TNBC [16, 17]. A phase II trial indicated that treatment with another antiandrogen, bicalutamide, resulted in a 19% clinical benefit rate in patients with AR+ TNBC [20]. Currently, enzalutamide is being examined in this same patient population alone (NCT01889238) and in combination with trastuzumab, the targeted anti-Her2 antibody, (NCT02091960) or taselisib, a PI3 kinase inhibitor, (NCT02457910).

Enzalutamide has been shown to be capable of a phenomenon called immunogenic modulation. Immunogenic modulation has been defined as the alteration of tumor cell phenotype in such a way that the tumor cell becomes more susceptible to immune-mediated cell death [21]. Therapies capable of inducing immunogenic modulation therefore have the potential to display synergistic therapeutic effects when combined with cancer immunotherapy [22]. Many conventional therapies used to treat cancer are capable of inducing immunogenic modulation. Chemotherapy, radiation and small molecule inhibitors can upregulate the expression of cell surface molecules such as tumor-associated antigens (TAAs), death receptors (ex. Fas), and major histocompatibility complex (MHC) moieties, making them better targets for immune-mediated attack [23–27]. More recently, however, studies have shown that ADT is also capable of immunogenic modulation [28, 29]. Enzalutamide was able to improve the sensitivity of human prostate carcinoma cell lines to cytotoxic T lymphocyte (CTL)-mediated killing *in vitro* through a novel form of immunogenic modulation, the alteration of apoptosis-associated gene expression [29].

Here we sought to investigate the effect of ADT, with enzalutamide or abiraterone, on human breast carcinoma cell lines to determine if ADT was capable of immunogenically modulating these cells. Our studies were able to show, for the first time, that ADT was capable of inducing immunogenic modulation in breast carcinoma cells and that, unlike what was previously seen with prostate carcinoma cells, this immunogenic modulation was not dependent on detectable AR expression. The immunogenic modulation induced by enzalutamide in breast cancer cells involved the modulation of both cells' surface tumor necrosis factor-related apoptosis-inducing ligand (TRAIL) receptor expression and apoptosis-associated gene expression. However, it was the alteration of expression of the anti-apoptotic gene, osteoprotegerin, that was critical for rendering breast cancer cells more sensitive to immune-mediated killing. Collectively, these data further support the use of ADT to treat AR+ TNBC and open up the possibility of also using it to treat AR negative (AR-) TNBC.

## RESULTS

### Enzalutamide reduced the proliferation of AR+ breast cancer cells

**Figure 1:**
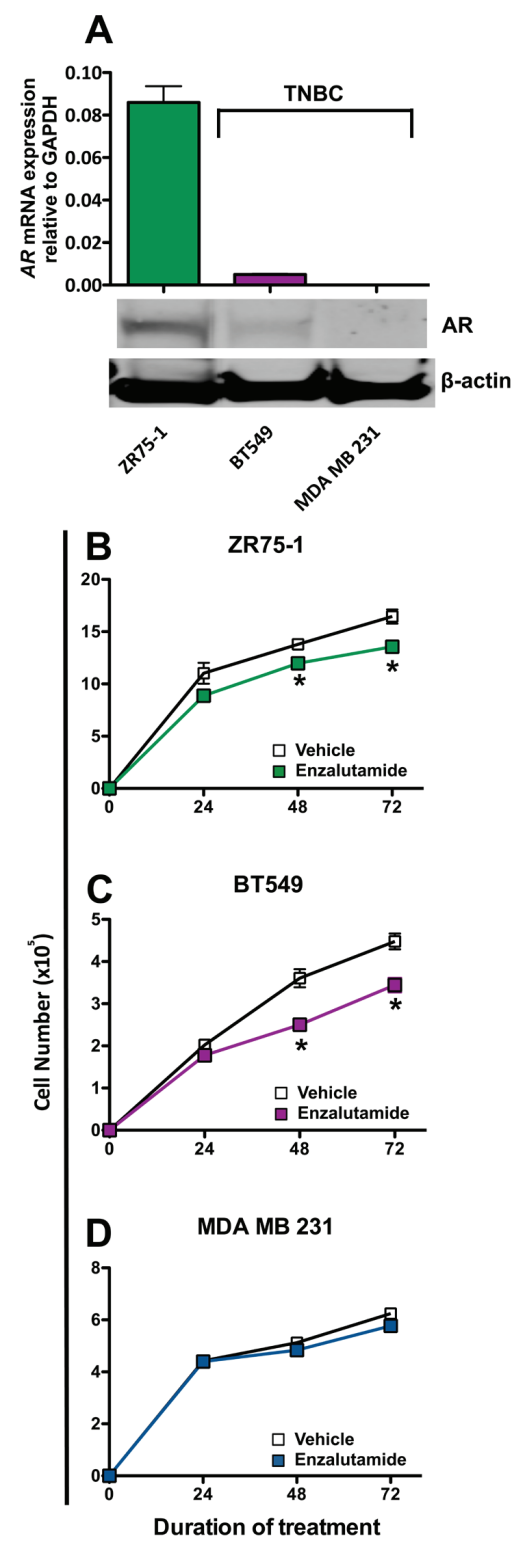
Enzalutamide inhibits the growth of androgen receptor (AR) positive breast cancer cells **A.** *AR* mRNA expression by ZR75-1, BT549 and MDA MB 231 cells as determined by quantitative RT-PCR and western blot. Breast cancer cells **B.** ZR75-1, **C.** BT549 and **D.** MDA MB 231 were exposed to enzalutamide (closed squares) or vehicle (DMSO, open squares) for 24, 48, and 72 hours, then assayed for growth and viability. Error bars indicate mean ± S.E.M. for quadruplicate measurements. Statistical analyses were done by Student's *t*-test, * = *P* < 0.05 *vs*. vehicle control. Data are representative of 3 independent experiments.

We chose to examine the effects of enzalutamide on breast carcinoma cells that represent three major classifications of breast cancer: luminal B (ZR75-1), mesenchymal-like (BT549) and mesenchymal stem-like (MDA MB 231). These cell lines also represent different combinations of estrogen receptor and androgen receptor positivity. ZR75-1 cells (ER+) also displayed a high degree of AR expression as determined by qRT-PCR and western blot, BT549 cells (ER-) expressed AR but at a much lower degree, and MDA MB 231 (ER-) cells did not express any detectable AR by quantitative real-time PCR or western blot analysis (Figure 1A). To determine the effect of enzalutamide on the proliferation of the breast cancer cell lines, each cell line was exposed to vehicle (DMSO) or 10 μM enzalutamide for 24, 48 or 72 hours. This level of exposure to enzalutamide mimics the clinically achievable median plasma concentration and was the dose shown to induce immunogenic modulation in prostate cancer cells [29, 30]. After the designated period of treatment, cells were harvested and counted, and their viability was measured by trypan blue exclusion. Enzalutamide significantly inhibited the proliferation of ZR75-1 (ER+AR+) cells (*P* < 0.05, Figure 1B) and to a greater degree that of BT549 (ER-AR+) cells (*P* < 0.01, Figure 1C) after 48 or 72 hours of treatment compared to vehicle control. However, despite this reduction, the cells continued to proliferate and their viability remained > 85% at all time points, regardless of treatment. Of note, despite large differences in AR expression levels between ZR75-1 and BT549 cells (Figure 1A), the degree of inhibition on cell proliferation mediated by enzalutamide was similar for both cell lines (1.2 fold and 1.3 fold reduction, respectively). Enzalutamide, however, had no effect on the proliferation of MDA MB 231 (ER-AR-) cells (Figure 1D), indicating that the presence of AR is required for the growth inhibitory properties of enzalutamide on breast cancer cells.

### Enzalutamide modulated the expression of tumor cell markers associated with immune recognition

**Figure 2:**
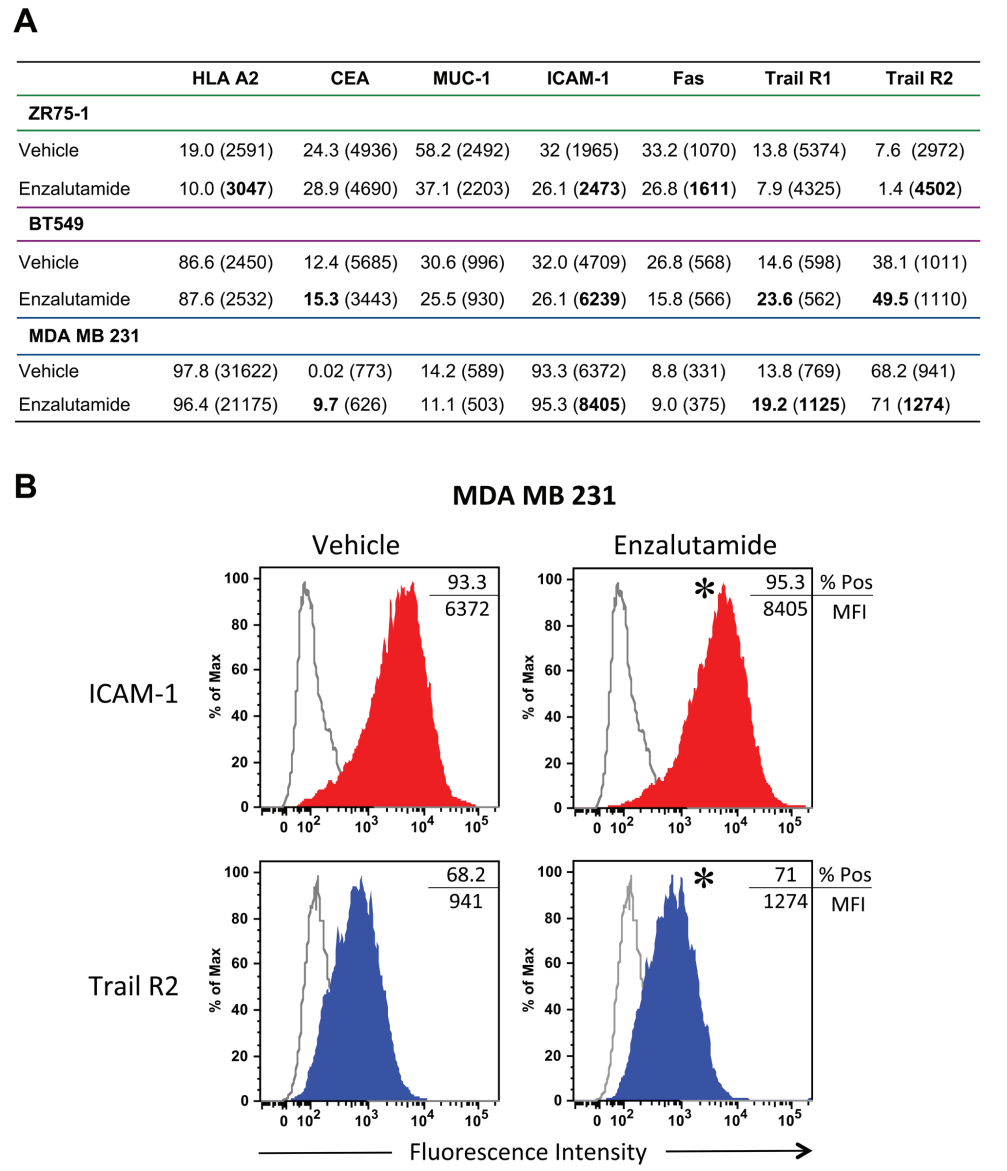
Flow cytometric analysis of surface marker expression on breast cancer cell lines exposed to enzalutamide ZR75-1, BT549 and MDA MB 231 cells were exposed to 10 μM enzalutamide for 48 hours then analyzed by flow cytometry for cell surface expression of HLA A2, CEA, MUC-1, ICAM-1, Fas, and tumor necrosis factor-related apoptosis-inducing ligand (TRAIL) receptors 1 and 2. **A.** Percent positivity and relative surface expression on a per-cell basis (mean fluorescence intensity (MFI)), in parentheses, are shown. Values in bold denote an increase of > 15% relative to vehicle-treated cells. Data shown is representative of experiment repeated 3 times with similar results. **B.** Representative histograms of ICAM-1 and Trail R2 expression for MDA MB 231. *p < 0.05.

It has been shown previously that radiation and chemotherapy can alter the cell surface phenotype of human tumor cells, rendering them more sensitive to T cell-mediated killing [25, 26]. To determine if enzalutamide could modify the expression of cell-surface markers that influence immune recognition, we treated the breast carcinoma cells with vehicle or 10 μM enzalutamide for 48 hours, then stained and analyzed them by flow cytometry. Treatment with enzalutamide significantly increased the expression levels (mean fluorescence intensity, MFI) of MHC-I, intracellular adhesion molecule 1 (ICAM-1), Fas and TRAIL receptor 2 on ZR75-1 (ER+AR+) cells (Figure 2A). However, this was combined with a concurrent reduction in the percent of cells displaying positivity. ZR75-1 (ER+AR+) cells also showed a reduction in mucin-1 (MUC-1) and TRAIL receptor 1 expression. Enzalutamide treatment upregulated the expression of ICAM-1 as well as TRAIL receptors 1 and 2 and the TAA carcinoembryonic antigen (CEA) on BT549 (ER-AR+) cells (Figure 2A). However, there was also a concurrent reduction in the expression level (MFI) of CEA as well as the percent of cells expressing MUC-1 and Fas. Interestingly, enzalutamide also significantly upregulated CEA, ICAM-1 and TRAIL receptors 1 and 2 on MDA MB 231 (ER-AR-) cells (Figure 2A) while concurrently reducing the expression levels of HLA A2 and MUC-1. Figure 2B displays representative histograms of ICAM-I expression for the 3 cell lines tested. Previous studies have suggested that improving the expression of any one of these markers could render tumor cells more amenable to T cell-mediated killing [23–26].

### ADT increased the sensitivity of breast cancer cells to immune-mediated lysis regardless of AR expression

**Figure 3:**
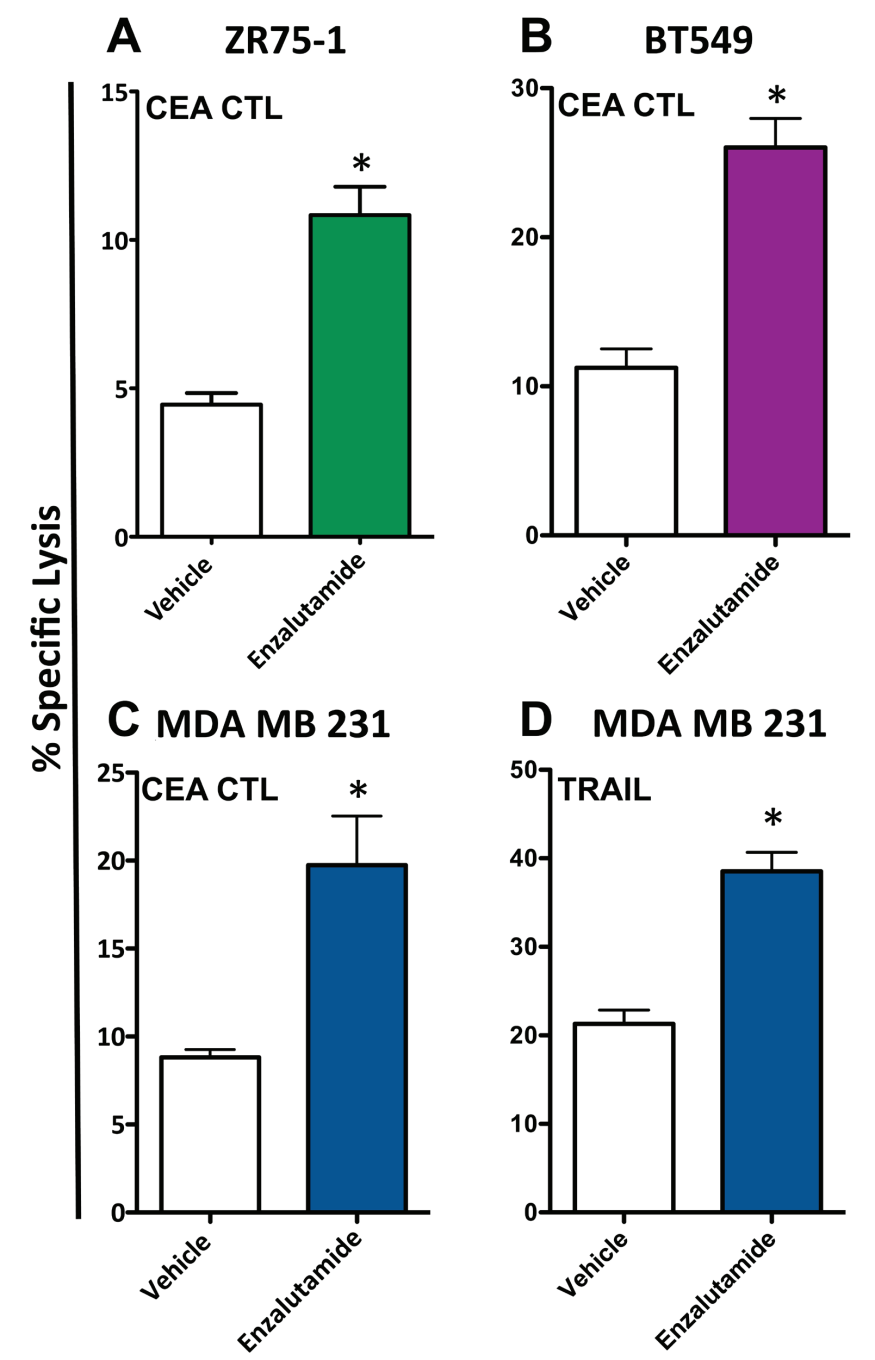
Enzalutamide increases the sensitivity of breast cancer cells to T cell- and TRAIL-mediated killing regardless of androgen receptor (AR) expression **A.** ZR75-1 (AR+, ER+), **B.** BT549 (AR+, TNBC) and **C.** MDA MB 231 (AR-, TNBC) cells were treated with either enzalutamide or vehicle, then used as targets in a CTL assay using CEA-specific CD8+ T cells as effector cells at an E:T ratio of 30:1. **D.** MDA MB 231 (AR-, TNBC) cells, treated with either enzalutamide or vehicle, were used as targets in a TRAIL-mediated lysis assay. Error bars indicate mean ± S.E.M. for quadruplicate measurements. Statistical analyses were done by Student's *t*-test, * = *P* < 0.05 *vs*. vehicle control. Data are representative of 2-4 independent experiments.

To determine whether enzalutamide was capable of increasing the sensitivity of breast cancer cells to antigen-specific T cell lysis, ZR75-1 (ER+AR+), BT549 (ER-AR+) and MDA MB 231 (ER-AR-) cells were treated with vehicle or 10 μM enzalutamide and used as target cells for CTL-mediated killing assays utilizing CEA-specific cytotoxic T-cells (CTL). Exposing ZR75-1 (ER+AR+) cells to enzalutamide significantly enhanced their sensitivity to CEA-specific CTL-mediated lysis compared to the vehicle control (*P* < 0.01, Figure 3A). Similarly, exposing BT549 (ER-AR+) cells to enzalutamide significantly improved their sensitivity to CEA-specific CTL-mediated lysis relative to vehicle-treated cells (*P* < 0.01, Figure 3B). However, enzalutamide was also capable of increasing the sensitivity of MDA MB 231 (ER-AR-) cells to CEA-specific CTL-mediated lysis compared to vehicle-treated tumor cells (*P* < 0.05, Figure 3C). Cytotoxic T cells can cause target cell lysis by multiple mechanisms including the release of perforin and granzyme, the binding of Fas ligand on the T cell to Fas on the target cell, and the binding of TRAIL on the T cell to TRAIL receptors on the target cell, all resulting in the induction of the apoptosis cascade. Both TNBC cell lines, BT549 (AR+) and MBA MB 231 (AR-), displayed an upregulation of TRAIL receptors in the absence of Fas upregulation (Figure 2A); thus we chose to further evaluate the effect of enzalutamide specifically on TRAIL-mediated lysis. To confirm the effect of enzalutamide on MDA MB 231 (ER-AR-) cells and to further investigate the effect of enzalutamide-induced TRAIL receptor upregulation, the cells were treated with vehicle or 10 μM enzalutamide and analyzed for their sensitivity to TRAIL-mediated lysis. Again, enzalutamide significantly improved the sensitivity of MDA MB 231 (ER-AR-) cells to TRAIL-mediated lysis (*P* < 0.01, Figure 3D). These results suggested that enzalutamide mediated immunogenic modulation in human breast carcinoma cells leading to their improved sensitivity to immune-mediated killing, and that this effect was independent of AR expression.

**Figure 4:**
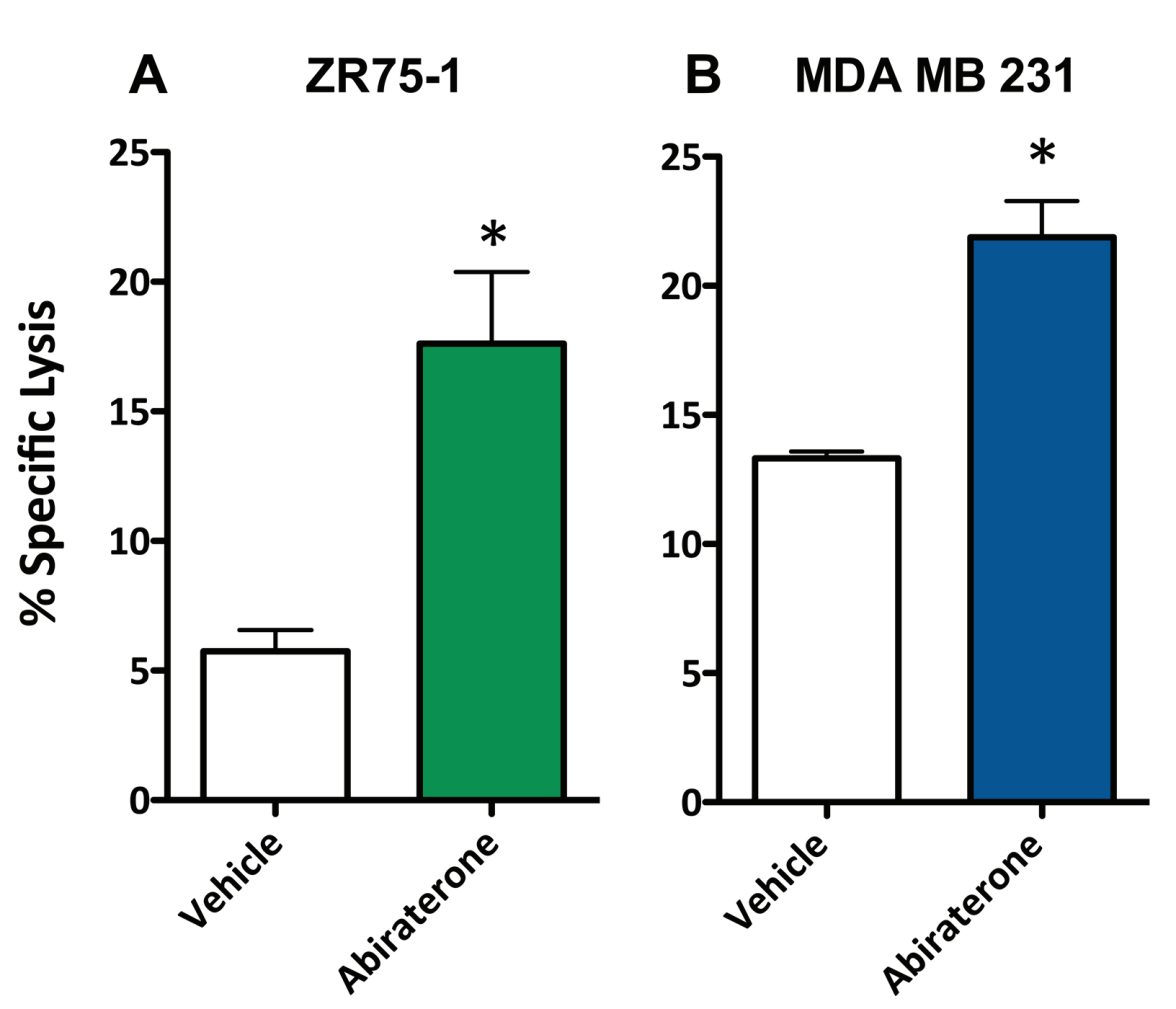
Abiratirone increases the sensitivity of breast cancer cells to T cell-mediated lysis regardless of androgen receptor (AR) expression **A.** ZR75-1 (AR+, ER+) and **B.** MDA MB 231 (AR-, TNBC) cells were treated with either abiraterone or vehicle, then used as targets in a CTL assay using CEA-specific CD8+ T cells as effector cells at an E:T ratio of 30:1. Error bars indicate mean ± S.E.M. for quadruplicate measurements. Statistical analyses were done by Student's *t*-test, * = *P* < 0.01 *vs*. vehicle control. Data are representative of 2 independent experiments.

To evaluate whether a second form of ADT was also capable of increasing the sensitivity of breast cancer cells to T cell lysis, 10 μM abiraterone or vehicle (DMSO) was used to treat ZR75-1 (ER+AR+) and MDA MB 231 (ER-AR-) cells. These cells were then used as target cells for CTL-mediated killing assays utilizing CEA-specific CTLs. As with enzalutamide, this concentration of abiraterone mimics the clinically achievable median plasma concentration and was the dose shown to induce immunogenic modulation in prostate cancer cells [29, 30]. Following treatment with abiraterone, ZR75-1 (ER+AR+) cells (*P* < 0.05, Figure 4A) and MDA MB 231 (ER-AR-) cells (*P* < 0.01, Figure 4B) both displayed enhanced sensitivity to CTL-mediated lysis compared to vehicle-treated cells. These results suggested that multiple types of ADT could successfully mediate improved immune-mediated lysis of human breast tumor cells regardless of their AR expression.

### Enzalutamide significantly reduces the expression of osteoprotegerin in AR-TNBC MDA MB 231 cells

**Figure 5:**
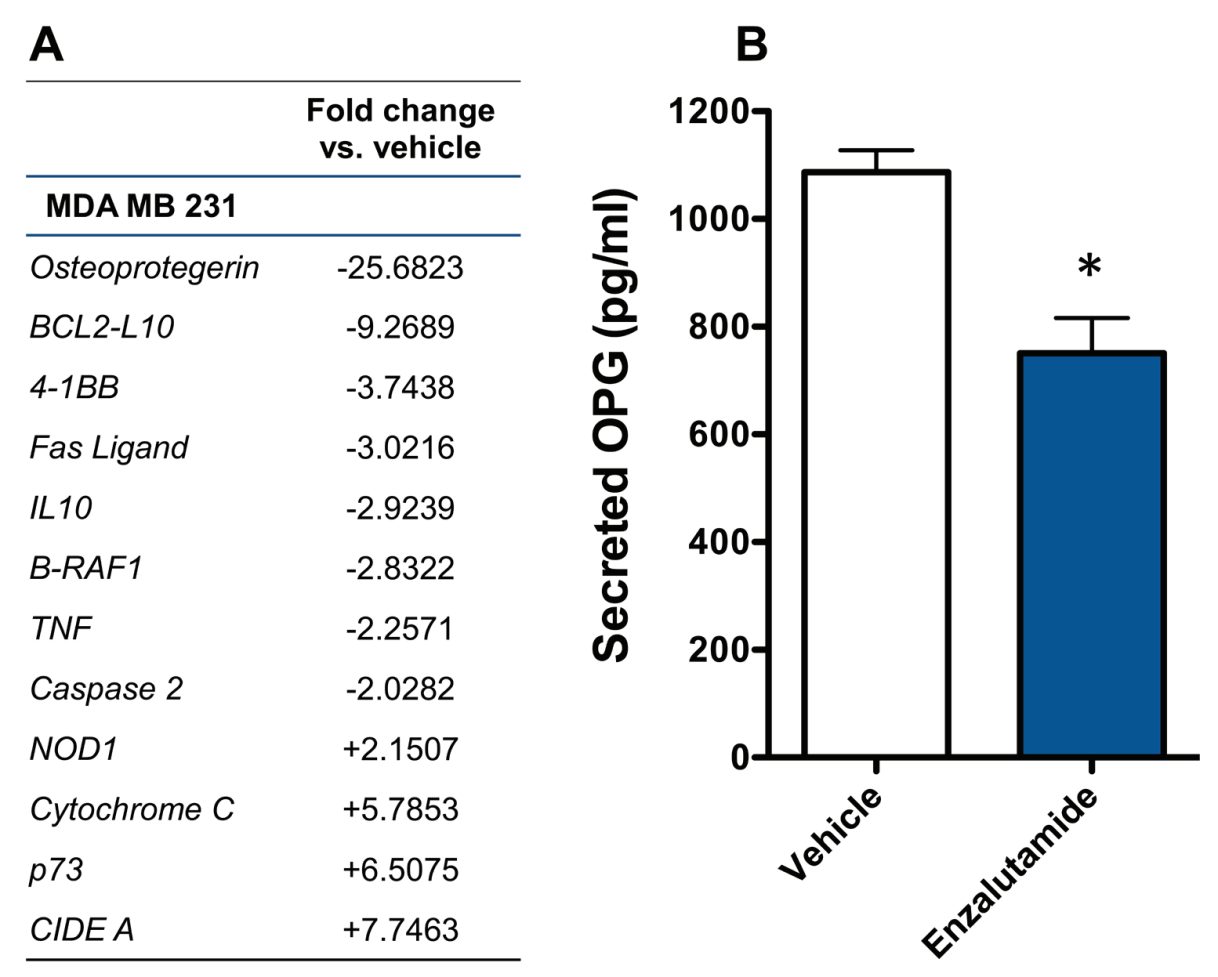
Enzalutamide significantly down-regulates the expression of osteoprotegerin (OPG) in MDA MB 231 (AR, TNBC) cells **A.** MDA MB 231 cells were treated with enzalutamide or vehicle for 24 hours. Changes of > 2-fold in apoptotic gene expression (mRNA) relative to vehicle control were determined by quantitative RT-PCR. **B.** MDA MB 231 cells were treated with 10 μM enzalutamide or vehicle for 48 hours. Levels of OPG in the supernatant of MDA MB 231 cells as determined by ELISA. Data are representative of 2 independent experiments.

Previously, enzalutamide has been shown to alter pro- and anti-apoptotic gene expression in prostate cancer cells [29]. We sougnt to examine whether enzalutamide was having a similar effect in breast cancer cells, which could play a role in its ability to increase their sensitivity to immune-mediated lysis. Here, we focused on MDA MB 231 cells, as these TNBC cells represented the cancer subtype with the most unmet clinical need. The mRNA expression of 90 genes involved in the apoptotic process was examined by qRT-PCR in enzalutamide-treated MDA MB 231 (ER-AR-) cells. Of these genes, 4 were up-regulated and 8 were down-regulated > 2-fold by enzalutamide treatment relative to the expression observed in vehicle-treated cells. Among these genes, *OPG*, an anti-apoptotic gene, was down-regulated ~25-fold by enzalutamide (Figure 5A). OPG is a secreted factor that belongs to the tumor necrosis factor receptor superfamily. We therefore sought to verify that this reduction in *OPG* mRNA resulted in reduced expression of OPG protein by ELISA. An ELISA for secreted OPG confirmed that 10 μM enzalutamide indeed reduced the amount of OPG expressed by MDA MB 231 (ER-AR-) cells (*P* < 0.05, Figure 5B).

### Modulation of OPG recapitulates the improvement in sensitivity to immune-mediated lysis observed in enzalutamide treated ER-AR- MDA MB 231 cells

**Figure 6:**
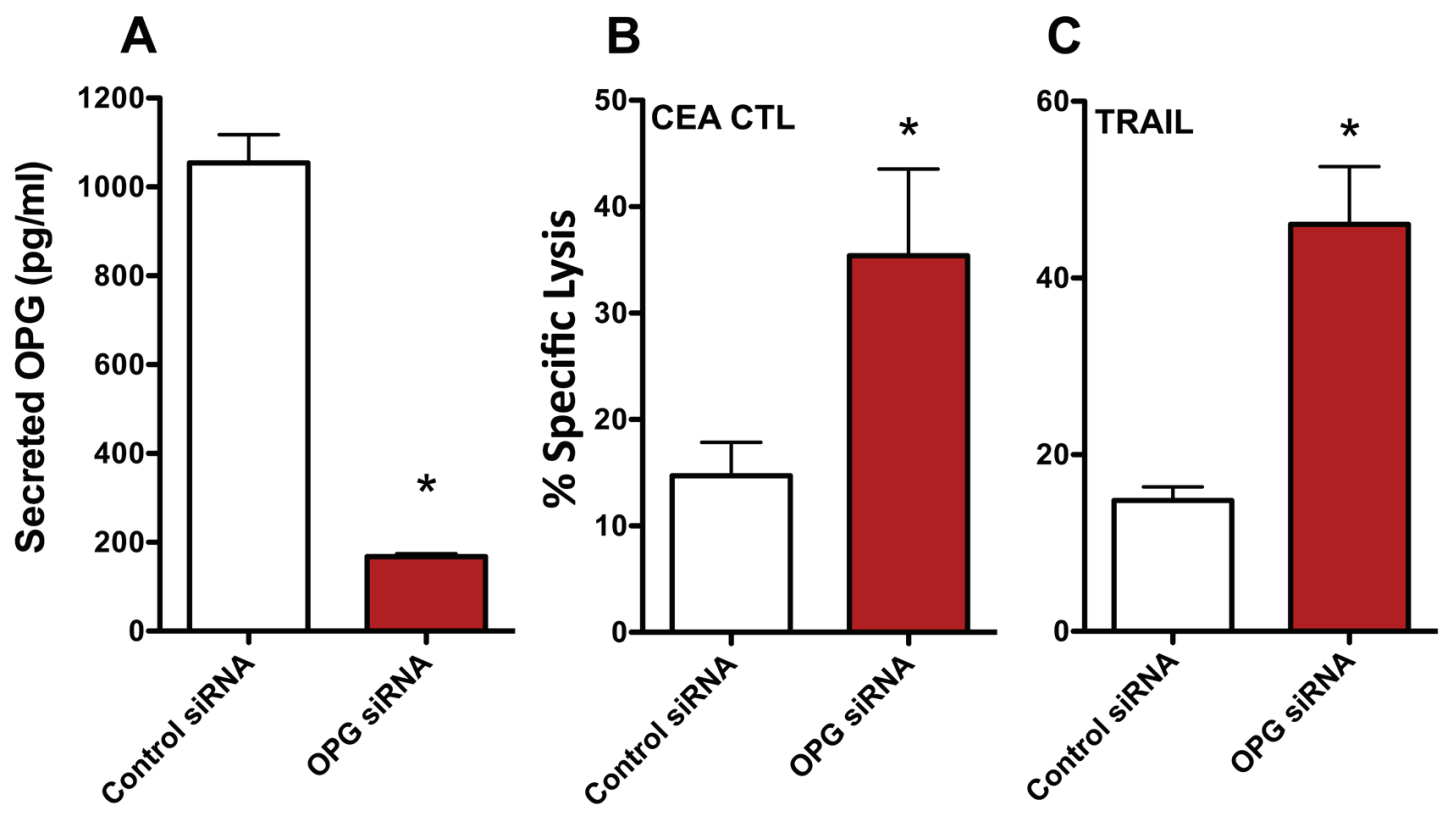
Knocking down osteoprotegerin (OPG) expression recapitulates the increased sensitivity of MDA MB 231 cells to T cell- and TRAIL-mediated killing MDA MB 231 (AR-, TNBC) cells were transfected with control or OPG siRNA. **A.** Amount of OPG in the supernatant of MDA MB 231 cells 48 hours after siRNA transfection. Sensitivity of MDA MB 231 cells to **B.** CEA-specific CD8+ T cell-mediated lysis or **C.** TRAIL-mediated lysis cells 48 hours after siRNA transfection. Error bars indicate mean ± S.E.M. for quadruplicate measurements. Statistical analyses were done by Student's *t*-test, * = *P* < 0.05 *vs*. vehicle control. Data are representative of 2 independent experiments.

To interrogate the role of OPG in the increase in CTL sensitivity mediated by enzalutamide, we transiently reduced the expression of OPG in MDA MB 231 (ER-AR-) cells using siRNA. An ~80% reduction in secreted OPG was confirmed by ELISA 48 hours post-OPG siRNA transfection relative to control siRNA transfected cells (Figure 6A). These MDA MB 231 (ER-AR-) cells were then evaluated for their sensitivity to CTL- and TRAIL-mediated killing. Similar to the results achieved with enzalutamide treatment, a reduction in OPG expression led to improved sensitivity of MDA MB 231 (ER-AR-) cells to both CEA-specific CTL-mediated lysis (*P* < 0.05, Figure 6B) and TRAIL-mediated lysis (*P* < 0.01, Figure 6C). These data suggest that the reduction in OPG expression played a major role in the increased sensitivity to immune-mediated killing that resulted from enzalutamide treatment.

## DISCUSSION

Until recently, the treatment of TNBC has not changed drastically over the past 20 years. For patients with breast cancer, first-line therapy (surgery, radiation and chemotherapy) is fairly equivalent among patients with receptor positive and TNBC. However, the lack of a tumor growth dependent target in TNBC patients most commonly results in ineffective management of disease in the adjuvant setting. Recently, however, studies have sought to further examine the molecular and cellular composition of TNBC in order to identify new therapeutic targets in this tumor type [4, 5, 7, 8]. The identification of a subset of TNBC that expresses AR and the subsequent studies that identified the AR signaling pathway as a growth stimulatory pathway in this subset of TNBC has stimulated interest in ADT as a potential treatment for this patient population [16, 17]. ADT is the standard of care for prostate cancer patients who have progressed following primary androgen ablation. Enzalutamide, a newly approved androgen deprivation agent, is an AR antagonist that blocks AR binding of androgens, prevents nuclear translocation of the ligand-receptor complex and inhibits DNA binding of the ligand-receptor complex [31]. Abiraterone, another form of ADT, inhibits CYP17A1, an enzyme required for androgen biosynthesis leading to reduced AR signaling as a result of a lack of androgens required to stimulate the signaling cascade [32]. These, as well as other ADT, are now being investigated for the treatment of breast cancer.

Enzalutamide has already shown antitumor efficacy against AR+ breast cancer in preclinical studies [16, 17]. In addition to blocking AR stimulated growth signals, however, a recent study suggested that enzalutamide also has additional anticancer properties. Ardiani, *et al*., observed that enzalutamide-treated prostate cancer cells were sensitized to immune-mediated lysis [29]. This effect was dependent on AR expression and was due to a reduction in the expression of the anti-apoptotic protein, neuronal apoptosis inhibitory protein. Additional studies have also shown that enzalutamide synergizes with cancer immunotherapy, improving overall survival in a mouse model of prostate cancer when combined with modified vaccinia Ankara and yeast-based cancer vaccines [28, 33]. Here we sought to determine if ADT, with enzalutamide or abiraterone, was capable of increasing the sensitivity of breast cancer cells to immune-mediated lysis and, if so, was this effect also dependent on AR expression.

We conducted our studies using 3 cell lines that represent 3 distinct types of breast cancer: ZR75-1 cells are luminal ER/PR+Her2+AR+ cells, BT549 cells are mesenchymal-like ER/PR-Her2-AR+ cells and MDA MB 231 cells are mesenchymal stem-like ER/PR-Her2-AR- cells. The AR expression of the cell lines used was confirmed by both RT-PCR and western blot (Figure 1A). There are contradictory reports on the expression of androgen receptor (AR) in the breast cancer cell line MDA MB 231. To further ascertain the gene expression levels of androgen receptor in these cells, we mined the expression levels from the NCI-60 Cell line Panel from Gene Expression Omnibus (NCBI) [34, 35]. Levels of androgen receptor were assessed in 5 breast cancer cell lines, BT-549, HS578T, MCF7, MDA MB 231, and T47D. There, MDA-MB-231 expressed the lowest levels of androgen receptor compared to the other 4 cell lines. These data confirm that of Hickey et al [36], who examined the levels of full-length AR in 10 breast cancer cell lines; MDA MB 231 expressed the second to the lowest levels detected, with the next highest expression in cell lines expressing 1.5 to 2 logs higher levels. Moreover, in looking specifically at AR-V7 splice variants in breast cancer cell lines, MDA MB 231 was the only cell line out of 10 analyzed that had no detectable copies of this variant. Seperately, Hu *et al*. [37], reported that MDA MB 231 expressed AR splice variants V1, V3, and V9 (but not V7), and virtually undetectable levels of full length AR. Barton *et al*. [17], showed very low levels of AR expression by western blot while Garay *et al*. [18], reported that these cells did not express AR by western blot analysis and that the AR gene had to be transfected into these cells to recapitulate growth inhibition by ADT. The MDA MB 231 cells used for the studies presented here were recently acquired from the ATCC and were shown to have undetectable levels of AR by both RT-PCR and western blot, even after long exposure (Figure 1A) [17, 18]. The ability of enzalutamide to reduce the growth rate of breast cancer cells has been shown to be strictly dependent on their expression of AR [16, 17]. Our data supported these observations as the growth of AR+ ZR75-1 (Figure 1B) and BT549 (Figure 1C) cells was inhibited by enzalutamide treatment, while the growth of AR- MDA MB 231 cells (Figure 1D) was unaffected. This is in contrast to the ability of enzalutamide treatment to increase the sensitivity of the cells to immune-mediated lysis. When enzalutamide-treated breast cancer cells were incubated with CEA-specific CTLs, their sensitivity to CTL-mediated lysis was improved regardless of AR expression, as the sensitivity of AR- MDA MB 231 cells (Figure 3C) was increased to the same extent as AR+ ZR75-1 (Figure 3A) and BT549 cells (Figure 3B). Variations in baseline lysis can be attributed to differing expression levels of CEA and HLA A2 (Figure 2A). The ADT drug abiraterone works by a different mechanism than enzalutamide; to confirm and extend our findings with enzalutamide, the ability of abiraterone to improve the sensitivity of breast cancer cells to immune-mediated lysis was also examined. As was observed with enzalutamide, abiraterone also increased the sensitivity of both AR+ ZR75-1 (Figure 4A) and AR- MDA MB 231 (Figure 4B) breast cancer cells to CTL-mediated lysis, confirming the ability of ADT to increase the sensitivity of breast cancer cells to immune-mediated killing regardless of AR status. Though abiraterone differs in its mechanism of action from that of enzalutamide, both therapies result in the reduction of androgen stimulation of the AR signaling pathway. Our data suggests that AR is not required for the immunomodulatory effect of enzalutamide, thus suggesting that this is an “off-target” effect. Abiraterone may be inducing immunogenic modulation through the same or a distinct mechanism. For example, we have not eliminated the possibility that our observations are due to an inhibition of androgen signaling through an alternate pathway which could be affected by both abiraterone and enzalutamide. The question of ADT therapy mediating immunomodulatory changes in cells lacking the target receptor is critical to the eventual clinical development of ADT as a therapy for TNBC and as such, future studies will focus on additional TNBC cell lines for molecular analysis. This suggests that ADT, especially when combined with cancer immunotherapy, may also provide therapeutic efficacy in TNBC patients who have the added negative prognostic indicator of being AR-. [10–12]. The increase in sensitivity of enzalutamide-treated MDA MB 231 cells to immune-mediated killing was also confirmed by using purified TRAIL (Figure 3D). TRAIL release is one mechanism by which T cells induce target cell apoptosis and TRAIL is also currently being examined as a novel cancer immunotherapeutic [38–40]. The increase in TRAIL-mediated lysis of enzalutamide-treated cells compared to vehicle-treated cells was very similar to that observed when CEA-specific CTLs were used, suggesting that enzalutamide treatment may be specifically affecting the tumor cell's sensitivity to TRAIL-mediated immune cell-induced lysis.

In addition to their cytotoxic and growth inhibitory effects, standard-of-care cancer therapeutics are able to alter the phenotype of tumor cells rendering them more sensitive to immune-mediated killing, a phenomenon termed immunogenic modulation. Therapies, such as radiation and chemotherapy, have exhibited the ability to induce immunogenic modulation by upregulating the expression of antigen presenting (MHC-I), adhesion (ICAM-1) and death receptor (Fas) molecules [23–25]. A similar pattern of cell surface receptor modulation was also observed in enzalutamide-treated breast cancer cells (Figure 2A). Among these was the upregulation of TRAIL receptor expression, particularly in the TNBC cell lines. This upregulation of TRAIL receptors could play a role in the observed increased sensitivity of the breast cancer cells to CTL- and TRAIL-mediated lysis (Figure 3). Due to the previously observed effects of enzalutamide on apoptotic gene expression [29], however, we chose to also examine apoptotic gene expression in enzalutamide-treated MDA MB 231 (ER-AR-) cells, as these cells represented the cancer subtype with the most unmet clinical need. The apoptotic gene array yielded a number of alterations in apoptotic gene expression between enzalutamide and vehicle-treated cells with the expression of anti-apoptotic gene *OPG* being down-regulated > 25 fold (Figure 5A). OPG is a decoy receptor for the receptor activator of nuclear factor (NF) kappa-B (kB) ligand (RANKL), which inhibits RANKL activation of the NF-kB transcription program. The main biologic function of OPG *in vivo* is to inhibit osteoclastogenesis during bone remodeling [41, 42]. By preventing the binding of RANKL, on the osteoblast, to RANK, on the immature osteoclast, OPG effectively disrupts osteoclast activation resulting in the inhibition of bone resorption [43–45]. However, OPG has also been shown to function as a soluble decoy receptor for TRAIL and inhibit TRAIL-mediated apoptosis [46, 47]. Particularly, OPG has been shown to inhibit the TRAIL-induced apoptosis of multiple tumor types, including breast cancer [48–50]. As OPG is a secreted factor, the enzalutamide-induced reduction in OPG was confirmed by ELISA (Figure 5B). Our data suggest that enzalutamide is able to reduce OPG expression in an AR-independent manner.

The role of reduced OPG in the enzalutamide-induced increase in breast cancer cell sensitivity to immune-mediated lysis was confirmed using siRNA knockdown studies. By significantly reducing the levels of secreted OPG (Figure 6A) we were able to recapitulate the increased sensitivity of MDA MB 231 (ER-AR-) cells to CTL- (Figure 6B) and TRAIL-mediated killing (Figure 6C). The observation that enzalutamide has no effect on the proliferation of MDA MB 231 cells (Figure 1D) in the presence of this significant reduction in OPG expression is not unexpected as these experiments were conducted in the absence of TRAIL. Taken together, these data support the conclusion that enzalutamide significantly reduces breast cancer cell expression of OPG and this reduced OPG expression leads to a significant increase in tumor cell sensitivity to immune-mediated lysis. Moreover, studies have linked OPG production to hormone signaling in osteoblasts, supporting the concept that inhibiting hormone signaling inhibits OPG expression [51, 52]. Clinically, a reduction in serum OPG has been observed in prostate cancer patients undergoing androgen ablation, again supporting the connection between hormone signaling and OPG production [53]. Although not examined here, this enzalutamide-induced reduction in OPG may also serve to inhibit breast cancer progression and metastasis as OPG has also been associated with increased breast tumor grade and metastatic potential [50, 54]. Together these data support the further investigation of enzalutamide-induced OPG reduction as a therapeutic modality for both AR+ and AR- TNBC patients.

Further subtyping of TNBC has yielded a subset of patients that may benefit from the tumor growth inhibitory effects of ADT. Here we have shown that ADT has substantial antitumor effects outside of its direct inhibition of AR signaling and that it may also benefit TNBC patients whose tumors do not express AR. By inducing immunogenic modulation in AR- breast carcinomas, particularly by reducing OPG expression, enzalutamide may not only provide monotherapy efficacy in this patient population but also could further impact TNBC patient care if combined with cancer immunotherapy.

## MATERIALS AND METHODS

### Tumor cells

ZR75-1 (CRL-1500), BT549 (HTB-122) and MDA MB 231 (HTB-26) breast cancer cells were purchased from American Type Culture Collection (Manassas, VA) in 2014 and were utilized at low passage number (< 5). All cells were maintained in RPMI-1640 medium supplemented with 10% fetal bovine serum, and 1% of HEPES, penicillin/streptomycin, L-glutamine, non-essential amino acids and sodium pyruvate. In addition, BT-549 cells required 10 μg/ml human insulin. All cells were regularly tested for and determined to be negative for *Mycoplasma* contamination and were discarded after 12 passages.

### Drug preparation

For *in vitro* studies, enzalutamide and abiraterone (Selleck Chemicals, Houston, TX) were dissolved in DMSO (vehicle, Sigma Aldrich, St. Louis, MO) to a concentration of 10 mM and stored at −20°C. A concentration of 10 μM of either enzalutamide or abiraterone was used for all *in vitro* experiments where media and drug or vehicle were replaced daily.

### RNA isolation, quantitative real-time PCR and apoptosis array

Quantitative real-time (RT) PCR was used to evaluate the *AR* mRNA expression levels of untreated ZR75-1, BT549 and MDA MB 231 breast cancer cells. Total RNA was isolated from the cells using the RNeasy Extraction Kit (Qiagen, Valencia, CA). RNA was reverse-transcribed into cDNA using the Advantage RT-for-PCR Kit (Clontech, Mountain View, CA). cDNA (10 ng) was used in a quantitative real-time PCR reaction using probes specific for *AR* (Hs00901571_m1) and *GAPDH* (4326317E) (Life Technologies, Grand Island, NY). *AR* mRNA expression level was calculated as expression relative to *GAPDH*. To evaluate the effect of enzalutamide on apoptosis-associated gene expression, MDA MB 231 cells were treated with either enzalutamide or vehicle for 24 hours. Total RNA was isolated from the cells using the RNeasy Extraction Kit. RNA was reverse-transcribed into cDNA using the RT^2^ First Strand Kit (SA Biosciences, Valencia, CA). Relative mRNA expression levels of 90 genes involved in apoptosis were assessed using an apoptosis PCR array (SA Biosciences) per the manufacturer's instructions. RT-PCR was performed on the 7300 Real-Time PCR System (Applied Biosystems, Carlsbad, CA).

### Western blotting

AR expression was confirmed by western blot using a rabbit monoclonal antibody to AR (Abcam, Cambridge, MA) and a mouse monoclonal antibody to β-actin (Cell Signaling, Danvers, MA). Untreated ZR75-1, BT549 and MDA MB 231 cells were lysed using Cell Lysis Buffer containing 1 mM PMSF (Cell Signalling, Danvers, MA) and 10 μL/mL HALT Protease/Phosphatase Inhibitor Cocktail (Thermo Scientific, Rockford, IL) according to the manufacturer's protocol. Protein concentration was measured using a BCA Protein Assay Kit (Thermo Scientific). Aliquots containing 50 μg of protein were run on a Bolt 4%-12% gradient Bis-Tris gel using the Bolt system, then transferred to a PVDF membrane using the iBLOT 2 Transfer System (Life Technologies). Membranes were blocked overnight at 4°C with PBS containing 5% BSA and 0.05% Tween20, then incubated with primary antibodies in block for 4 hours at room temperature. Membranes were then incubated with IRDye-labeled goat anti-rabbit and goat anti-mouse secondary antibodies (LI-COR Biosciences, Lincoln, NE) at a 1:10000 dilution in block for 1 hour at room temperature. Membranes were imaged using the Odyssey Infrared Imaging System (LI-COR Biosciences).

### Tumor cell proliferation

To evaluate the effect of enzalutamide mediated androgen deprivation therapy on breast cancer cell proliferation, ZR75-1, BT549 and MDA MB 231 cells were treated with either enzalutamide or vehicle (DMSO) for 24, 48, or 72 hours. At the indicated time points, cells were harvested and the number of viable cells was determined by trypan blue exclusion.

### Flow cytometry

To assess the effect of enzalutamide on the cell surface phenotype of breast cancer cells, ZR75-1, BT549 and MDA MB 231 cells were treated with either enzalutamide or vehicle for 48 hours. After 48 hours, cells were harvested and stained with the following antibodies: HLA A2-PE-Cy7 (MHC-I), MUC-1-FITC (TAA), CD54-BV421 (ICAM-1), CD95-FITC (Fas) (BD Biosciences, San Jose, CA), CEA-APC (TAA) (Miltenyi Biotec, Auburn, CA), TRAIL receptor 1 and TRAIL receptor 2 (R & D Systems, Minneapolis, MN). LIVE/DEAD Fixable Violet Dead Cell Stain (Life Technologies, Grand Island, NY) was used to determine cell viability. Cells were incubated with the antibodies for 30 min at 4°C, acquired on a FACS Verse flow cytometer (Becton Dickinson, Franklin Lakes, NJ), and analyzed using FlowJo software (TreeStar, Inc., Ashland, OR).

### Cytotoxic T lymphocyte and TRAIL killing assays

To determine the ability of ADT to alter the sensitivity of ZR75-1, BT549 and MDA MB 231 cells to CTL- or TRAIL-mediated lysis, cells were treated with enzalutamide, abiratirone, or vehicle for 48-72 hours, after which they were harvested, washed, and used as targets in standard lysis assays. Here, live tumor cells are metabolically labeled with in-labeled oxyquinoline (Medi-Physics Inc., Arlington Heights, IL) and coincubated in 96-well round-bottom plates at 37°C/5% CO2 with HLA-A2-restricted CEA-specific CTLs at an effector:target ratio of 30:1 or 500ng/ml KillerTRAIL (Enzo Life Sciences, Farmingdale, NY). The HLA-A2-restricted CEA-specific CTL recognizes the CEA peptide epitope YLSGANLNL (CAP-1), and has previously been described [55]. After 18 hours, supernatants were harvested and analyzed for the presence of in using a WIZARD2 Automatic Gamma Counter (PerkinElmer, Waltham, MA). The percentage of tumor lysis was calculated as follows: % tumor lysis = [(experimental cpm - spontaneous cpm) / (maximum cpm - spontaneous cpm)] × 100.

### ELISA

The level of secreted OPG was confirmed in MDA MB 231 cells treated with 10 μM enzalutamide or vehicle for 48 hours using a DuoSet ELISA (R & D Systems) according to the manufacturer's instructions. ELISA was performed on combined supernatant samples taken following 24 and 48 hours of treatment.

### RNA interference (siRNA)

OPG expression was inhibited in MDA MB 231 cells using siRNA duplexes targeting OPG sequences and control siRNA duplexes (Origene, Rockville, MD). MDA MB 231 cells were transfected with OPG or control siRNA according to the manufacturer's instructions. The interference of OPG expression was confirmed 48 hours post siRNA transfection by ELISA as described. Forty-eight hours post siRNA transfection, the MDA MB 231 cells were also used as target cells in CEA-specific CTL- and TRAIL-mediated lysis assays as described.

### Statistical analysis

GraphPad Prism 5 statistical software (GraphPad Software, La Jolla, CA) was used to measure 2-tailed unpaired Student's t tests for differences between groups, with a 95% confidence interval. All data represent the mean ± SEM. for the indicated number of replicates. FlowJo software was used to determine significant differences in the distribution of flow cytometry data using the Kolmogorov-Smirnov test.

## Abbreviations

TNBC: triple-negative breast cancer
AR: androgen receptor
TRAIL: tumor necrosis factor-related apoptosis-inducing ligand
OPG: osteoprotegerin
ER: estrogen receptor
PR: progesterone receptor
Her2: Her2/neu receptor
ADT: androgen deprivation therapy
FDA: Food and Drug Administration
TAA: tumor associated antigen
MHC: Major histocompatibility complex
CTLs: cytotoxic T lymphocytes
DMSO: dimethyl sulfoxide
qRT-PCR: quantitative real-time polymerase-chain reaction
mRNA: messenger RNA
HLA: Human leukocyte antigen
PE-Cy7: tandem fluorochrome combining PE and the cyanine dye Cy7
MUC-1: Mucin 1, cell surface associated (MUC1) or polymorphic epithelial mucin
FITC: fluorescein isothiocyanate
ICAM-1: Intercellular Adhesion Molecule
CEA: carcinoembryonic antigen
APC: allophycocyanin
siRNA: small interfering RNA
SEM: standard error of the mean
KS: Kolmogorov-Smirnov test
MFI: mean fluorescent intensity
